# Use of UV Treated Milk Powder to Increase Vaccine Efficacy in the Elderly

**DOI:** 10.3389/fimmu.2018.02254

**Published:** 2018-10-17

**Authors:** Sara Schaefer, Kasper Arthur Hettinga, James Cullor, J. Bruce German, Bethany M. Henrick

**Affiliations:** ^1^Department of Food Science and Technology, Foods for Health Institute, University of California, Davis, Davis, CA, United States; ^2^Wageningen University and Research, Wageningen, Netherlands; ^3^School of Veterinary Medicine, University of California, Davis, Davis, CA, United States; ^4^Food Science and Technology Department, University of Nebraska Lincoln, Lincoln, NE, United States

**Keywords:** nutritional supplementation, dairy proteins, immune response, vaccines, UV-C treatment

## Abstract

Aging populations experience a decline in adaptive immune system function also known as immunosenesence. Protein nutrition has been shown to stimulate and strengthen the immune system, and such approaches are needed for this growing segment of the population. A controlled, randomized, double blind pilot study was conducted to compare two different protein sources (soy and dairy) as nutritional supplementation to enhance vaccine response. Our objective was to examine the immune stimulating effects of dairy protein subjected to ultraviolet radiation (UV-C) radiation treatment process instead of pasteurization. Participants were 21 healthy individuals over 60 years of age who consumed 6 g of the dairy protein or a comparison, soy isoflavone protein, twice a day for 8 weeks. DTaP vaccine administered at week 4. Non-parametric *t*-tests revealed a significant increase in Tetanus antibodies in the dairy group compared to the soy group at week 8. These findings suggest additional benefits of UV-C treated unheated dairy protein as a solution to counteract immunosenescence, but warrant further study in elderly and other populations that might benefit from immune system stimulation.

## Introduction

Immunization is an effective strategy against infectious disease; however, immunological function often falters as humans age (1, 2). Data indicate that 90% of deaths from infectious disease in the elderly are vaccine-preventable (3). Immunosenescence is a condition in which the immune system decreases in its response to pathogens. Likewise, immunosenescence often decreases the efficacy of vaccines in the elderly, increasing this particular demographics' susceptibility to severe infection (4). Several reports have demonstrated that older individuals lack robust response to conventional vaccines (4–6). For example, antibody responses to influenza and tick-borne encephalitis vaccine were impaired in the elderly (5). Such data suggest that immunosenescence is an important issue to overcome in the development of effective vaccine response in aging populations and for improving immune function in general. Taken together with the increased proportions of persons living beyond 65 years of age (7), these data indicate a need for alternative strategies to decrease immunosenescence, and thereby enhance immune responses in aging populations.

Protein-energy malnutrition has been linked with impaired immune function in the elderly (2, 8). Protein supplementation can enhance the immune system response, and there is evidence that dairy protein may yield additional benefits over plant-based protein sources, (9). While these data are promising, more research is needed to confirm the immune benefits of dairy protein over non-dairy sources, and also the impact of the various sources of dairy protein.

While raw dairy has been associated with notable risks caused by ingestion of milk borne pathogens, it is also associated with several health benefits including decreased incidence of asthma, allergies and respiratory infections (10). This may suggest some immune benefits of dairy proteins are lost upon heat processing. Now, non-thermal processing technologies can render milk safe by reducing microorganisms to a similar level as milk subjected to a thermal pasteurization process, while at the same time aiming to retain the bioactivity of the dairy proteins. TruActiv™ NF is a protein product isolated from bovine milk that is manufactured in the U.S. It uses ultraviolet radiation (UV-C) treatment of a raw nonfat milk to destroy or eliminate the most resistant microorganisms of public health concern, based on previously described enumeration tests (11–14). Hence, UV-C treatment of raw milk allows for a method other than heat pasteurization to destroy or eliminate the most resistant microorganisms of public health concern, as discussed in the Food, Drug, and Cosmetic Act (FD&C, 2013) (15). Several studies have concluded UV-C treatment to be an effective technology to reduce microbial populations in milk and other foods (16–18). It is yet unknown however, whether UV-C treated milk protein has immunological benefits.

More research is needed to understand the general benefits of dairy products on immune stimulation as a potential strategy to counteract immunosenescence in the elderly. The goal of the present study is to evaluate the impact of dairy protein supplementation on vaccine response by using a novel and safe unheated dairy protein source. We examined DTaP vaccine response in two elderly group's supplemented with either the novel dairy protein or soy protein. The study objectives were to (1) characterize and quantify DTaP-specific antibodies from the peripheral blood of participants, and (2) determine how consumption of the supplements correlate with specific antibody response.

## Materials and methods

### Recruitment and screening

A controlled, randomized, double blind pilot study was conducted that measured DTaP vaccine response in two groups of elderly volunteers (*n* = 21) supplemented with either the dairy or soy protein. Healthy volunteers were recruited from retirement centers and long-term care facilities in Northern California. The DTaP vaccine was selected since Pertussis is of growing concern in the elderly; however, it is not currently required for admission to these facilities.

*Inclusion criteria* included participants be more than 60 years of age. Volunteers underwent a physical examination and health assessment by a physician to ensure the absence of *exclusion criteria* which were regular consumption of greater than one unit of milk and/or milk products (milk, yogurt, fresh cheese, etc.) a day at the time of enrollment, known milk allergy, food faddism, other non-traditional diet, prolonged consumption of dairy supplements (greater than one daily during the previous four weeks), use of tobacco products in the previous 10 years, underlying neoplasia or immunological disease, including hypergammaglobunemia, renal disease or failure, use of steroids or immunosuppressive drugs in the previous eight weeks, reduced physical activity (New York Heart Association classes III-IV). Having received a DTaP vaccine within the last 5 years was an exclusion criteria for the study, but patient records were incomplete for many volunteers on this aspect. Therefore, volunteers with an initial Tetanus antibody level above 3 IU/mL were excluded from the study results as the volunteer was assumed to have had the DTaP vaccine within the last 5 years. Each volunteer was offered a $50 gift certificate to CVS Pharmacy upon completion of the study. All eligible participants were enrolled and signed the consent form approved by the local Institutional Review Board. This trial is registered at ClinicalTrials.gov as NCT03557463.

### Human samples

**Figure 1:**
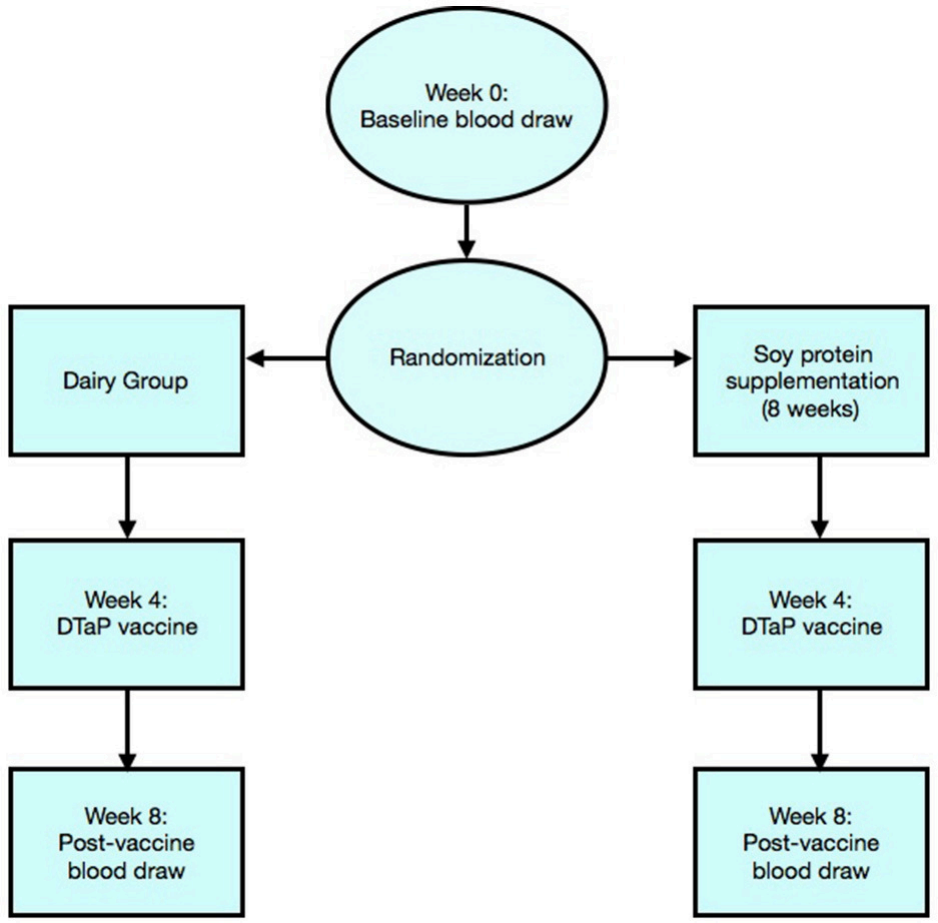
Flowchart illustration represents randomization of participants and progress through the phases of the trial.

Peripheral blood samples were collected at the time of enrollment (week 0) and serum was stored at −80°C until subsequent analysis. Participants were then randomized into two groups and provided with equal concentration and quantity of either dairy or soy supplement provided in powdered form in coded, single-serving bags. Both participants and researchers were blinded to the type of protein received. Participants were asked to consume two servings of protein powder (6 grams/packet) with 4 ounces of water or applesauce twice per day, with meals, for a total of 8 weeks. At week 4, participants were vaccinated with DTaP vaccine (Sanofi Pasteur Inc., Swiftwater PA). A 0.5-mL dose of Adacel® is formulated to contain 5 Lf of tetanus toxoid, 2.5 Lf of diphtheria toxoid, 8 μg of inactivated PT, 8 μg of FHA, and 2.5 μg of pertactin (69 kiloDalton outer membrane protein). Each 0.5-mL dose contains aluminum hydroxide as adjuvant (not more than 0.39 mg aluminum by assay), 4.5 mg of sodium chloride, ≤ 100 μg of residual formaldehyde, and ≤ 100 μg of polysorbate 80 (Tween 80). A second blood draw was obtained 4 weeks after vaccination (week 8) and serumcollected for DTaP antibody analysis. The design and participant progression through the study is presented in Figure 1.

### Protein supplements

Low isoflavone soy protein was purchased commercially from ADM, Minneapolis, USA. Tamarack Biotics, LLC provided a UV-C treated raw milk protein supplement, TruActiv MPC 85. Briefly, raw milk is exposed to UV-C light in a turbulent flow system at a rate of 4,000 L/hour with an applied UV-C dose of 2,000 J/L. After UV-C treatment, the milk was dried using a high volume air dryer with maximum temperature exposure of 42°F (6°C) for 2 min, packaged into 25 kg aluminum storage bags with oxygen scavenger and stored frozen. Prior to participant consumption, the batch of UV-C treated milk powder was analyzed by third-party National Food Laboratory, LLC per the following minimum guidelines, as stipulated by the FDA Pasteurized Milk Ordinance (19).

Table 1 contains the analysis of one dose of the dairy protein source present in one serving of the powdered supplement consumed by participants. Each participant was required to consume a total of 112 servings for the 8-week duration of the study. A 4-week supply was provided at baseline (week 0) and at the time of vaccine administration (week 4). Six grams of either the dairy or soy protein and 2 grams of a flavoring ingredient (vanilla flavored powder) were measured into single serving bags. Bags were coded based on protein type and both participants and researchers were blinded to the underlying code. All subjects were instructed to prepare and consume the supplement with water or added to applesauce, twice a day in addition to their normal diet. Participants were explicitly advised not to use the supplement as a substitute for any part of their normal diet. Compliance was evaluated by the assisted living facility nursing staff throughout the study by collection of sample containers.

**Table 1:** Product Analysis of TruActiv MPC85.

**Analytical Test**	**Results**		**Specifications**		**Test Method**
Standard plate	8,500	CFU/g	<10,000	CFU/g	USP <2021>
Coliform count	<3	MPN/g	<2,500	MPN/g	AOAC 966.24
*Escherichia coli*	<3	MPN/g	<3	MPN/g	AOAC 988.19
*Staphylococcus*	Absent	Per 10 g	Absent	Per 10 g	USP <2002>
*Salmonella*	Absent	Per 25 g	Absent	Per 25 g	USP <2002>
Yeast	<100	CFU/g	<5,000	CFU/g	USP <2002>
Mold	<100	CFU/g	<5,000	CFU/g	USP <2002>
*Listeria*	Negative	Per 25 g	Negative	Per 25 g	FDA BAM
Moisture	5.37	Wt. %	<7	Wt. %	AOAC 930.15

### Measurement of antibody response

Serum samples were obtained at week 0 and week 8, stored at −80° C and analyzed for concentration of DTaP-specific antibodies using Enzyme Immunoassay (EIA) (Quest Diagnostics Infectious Disease, Inc., San Juan Capistrano, CA). Type-specific antibody concentration post-vaccine ([Ab_8_]_Tx_) minus the concentration pre-vaccine ([Ab_0_]_Tx_) was used to determine the change in antibody concentration for a given serotype (D[Ab]_Tx_).

Results were expressed quantitatively (μg/mL) based on the obtained standard curves. Samples included all individuals who completed the supplementation trial with the dairy (*N* = 10) and soy protein (*N* = 11). A minimal four-fold increase between pre-immunization and post-immunization sera is considered a normal response to Tetanus toxoid. Levels >0.50 IU/mL are generally considered protective, whereas levels between 0.05 and 0.49 IU/mL are indeterminate for the presence of protective antibody and may indicate a need for further immunization to Tetanus toxoid.

### Statistical analysis

Data between different groups were compared using the nonparametric Mann–Whitney *U*-test (continuous variables) or chi square test (categorical variables). Nonparametric tests were used due to the small sample size. Mean and standard error of the mean (SEM) are reported for the change in antibody response for each volunteer. The average change in antibody concentration within the dairy and soy groups were compared. For each antibody, data between groups were compared using nonparametric *t*-tests for continuous variable and chi square test for categorical variables. All analyses were two-tailed and *p* < 0.05 considered statistically significant. Analyses were conducted in SPSS (Version 24) for Mac OSX.

## Results

A total of 21 participants aged 63-94 years (average age 73.4 ± 9.2) were included in final analyses (Table 2). Of 38 participants originally enrolled, 8 dropped out (2 due to intestinal distress, 3 did not like the taste, 2 had low compliance, and 1 for unknown reasons). Nine volunteers were excluded who had a baseline Tetanus antibody level greater than 3 IU/mL and so assumed to have received DTaP vaccine within 5 years.

**Table 2:** Demographic and health characteristics of two study groups.

	**Dairy protein (*N =* 10)**	**Soy protein (*N* = 11)**
Age (years)^*^	72.30 ± 8.04	74.36 ± 10.49
Male (N)	5	4
Female (N)	6	6
Body Mass Index (kg/m^2^)^*^	28.67 ± 4.25	28.47 ± 3.00
Exercise (days/week)^*^	2.21 ± 2.55	2.86 ± 2.56

* *Continuous variables are expressed as mean ± SEM. Values are not significantly different between groups*.

Tetanus antibody levels were not different between supplementation groups at week 0. Both supplementation groups exhibited an increase in Tetanus antibody levels at 4 weeks post vaccine. Nonparametric *t*-tests (Mann–Whitney U) revealed a significantly greater increase in Tetanus antibody levels in the group that received the UV-C treated dairy protein versus the soy protein supplement (11.20 ± 1.05 versus 7.10 ± 1.34 IU/mL; *p* = 0.029), see Table 3. Response to diphtheria and pertussis (PT and FHA) vaccines were also greater in the dairy protein group, but differences were not statistically significant.

**Table 3:** Change in Tetanus antibody concentration (IU/mL) in two elderly groups supplemented with the dairy and soy protein.

**Soy**	**Tetanus Antibodies (IU/mL)**		
	**Week 0**	**Week 8**	**Week 8 - Week 0**
1	0.65	3.60	2.95
2	2.73	6.26	3.53
3	0.64	7.73	7.09
4	0.85	5.14	4.29
5	0.69	16.00	15.31
6	0.80	12.98	12.18
7	2.31	7.66	5.35
8	2.37	15.70	13.33
9	1.20	4.59	3.39
10	1.09	4.75	3.66
11	1.35	8.32	6.97
Mean ± SEM	1.33 ± 0.23	8.43 ± 1.35^**^	7.10 ± 1.34^*^
**Dairy**	**Week 0**	**Week 8**	**Week 8 - Week 0**
1	1.01	6.58	5.57
2	0.91	11.90	10.99
3	0.30	11.90	11.60
4	1.54	10.70	9.16
5	0.88	8.84	7.96
6	1.22	16.00	14.78
7	0.59	9.62	9.03
8	1.02	16.00	14.98
9	0.73	13.00	12.27
10	0.38	16.00	15.62
Mean ± SEM	0.86 ± 0.12	12.05 ± 1.03^**^	11.20 ± 1.05^*^

* *Average change in antibody levels (week 8 - week 0) is significantly different between groups (p = 0.029)*.

** *Average post-vaccine antibody level is higher at week 8 in dairy group compared to soy group (p = 0.034)*.

*A multiple linear regression was conducted to explore the relationship between antibody level changes and volunteer age and sex and no effects were seen*.

## Discussion

The results of this randomized, double-blind pilot study suggest that consuming a nutritional supplementation from UV-C treated unheated dairy protein may have increased benefits for stimulating the immune system in senior citizens. For generalizability, these preliminary results should be replicated in a larger population. Future research should also include a group consuming heat-treated milk to determine whether the benefits are associated with the absence of heating in the UV-C treated milk protein.

Immunosenescence is a significant concern in aging populations accounting for higher incidence of infection and poor vaccine response (1, 2). The aging immune system undergoes changes that impair adaptive immune response (20). Diet-based approaches have potential to boost immune stimulation and counteract immunosenescence. A significant portion of the immune system is located in the mucosal lining of the digestive tract. Our results suggest that unheated dairy protein was associated with enhanced immune response to the tetanus vaccine. Our results are in agreement with (9) who demonstrated an enhanced response to a *Streptococcus pneumoniae* vaccine among elderly supplemented with whey protein compared to those who were supplemented with soy protein.

Because in the Freeman study it was demonstrated that heated whey proteins already benefit immunological response, we can reasonably anticipate that the difference between a heated and unheated milk group would be smaller. Because the present study was a small pilot, it would not likely carry sufficient statistical power to detect a difference between heated and unheated milk proteins. Rather, heated milk proteins should be included in a larger follow-up study that allows for sufficient power. Very few studies have examined the differences between unheated and heated milk proteins, although one group demonstrated increased immune system activation in a mouse model fed raw milk protein compared to a heated milk protein (21).

Brick (22) showed that with increasing heat intensity, more immunologically active milk proteins become damaged. It is reasonable to hypothesize that the effect seen in the mouse study by (21) was caused by to damage to the immunologically active proteins in the heated milk protein, as milk protein is the most heat sensitive fraction. Proteomics analyses carried out similar to (22) show that the typical heat damage can be prevented by applying UV-C to milk (data not shown). This indicates that the effect of heating shown by Brick et al. (22) may extend to powdered milk protein products.

These hypotheses are further supported by studies that have shown raw bovine milk to be immunologically supportive, aiding in the prevention of allergy, asthma and respiratory illness in children (23–26). Wyss et al. (27) reported raw milk consumption in childhood to be associated with higher pulmonary function that lasts into older adulthood. Until now, the risks of raw milk including food borne illness and pathogens have far outweighed the benefits (28). Non-thermal UV-C radiation techniques offer a solution by eliminating illness causing microorganisms in a capacity similar to thermal processing techniques. To our knowledge this is the first study to examine the impact of UV treated milk protein on immune response in any population. There have been no studies to show immune enhancing benefits over pasteurized dairy sources.

Because this was a small pilot study, results are limited in generalizability and larger clinical studies are needed to confirm immune supportive effects of dairy protein derived from UV-C treated raw milk. In addition to small sample size, this study has several other limitations. Although the age range of elderly volunteers varied widely (60–94 years) it was not possible to stratify by age in the analyses due to a limited power but the authors recommend follow-up trials incorporate age stratification to determine differences between age groups. The nutritional status of participants was not assessed due to lack of resources available in this preliminary study. However, due to the confounding relationship with immune function, nutrition status should be accounted for in this population (9). Although it is worthwhile to note that participants were likely consuming somewhat similar diets because they consumed food from the same assisted living center.

## Conclusion

In conclusion, this pilot study demonstrated a potential immune stimulating effect of UV-C treated raw dairy protein in vaccine response among elderly participants. These early findings suggest there may be additional benefits of UV-C treated raw dairy proteins for immunosenescence, but warrant further study in elderly and other populations that can benefit from immune system stimulation.

## Ethics statement

This study was carried out in accordance with the recommendations of WIRB. The protocol was approved by the WIRB. All subjects gave written informed consent in accordance with the Declaration of Helsinki.

## Author contributions

SS is the primary author and performed data analyses and primary writing of the manuscript. KH contributed to manuscript writing and revision. JC contributed to manuscript writing and revision. JG contributed to the study design, manuscript writing and revision. BH designed the study and contributed to manuscript writing and revision.

### Conflict of interest statement

SS has received consulting fees from Tamarack Biotics for scientific analyses. KH is a general advisor on milk composition & milk processing to Tamarack Biotics and has performed analytical services. BH previously received consulting fees from Tamarack Biotics for study design and contributions to writing the manuscript. The remaining authors declare that the research was conducted in the absence of any commercial or financial relationships that could be construed as a potential conflict of interest.
