# Corrigendum: Use of UV Treated Milk Powder to Increase Vaccine Efficacy in the Elderly

**DOI:** 10.3389/fimmu.2019.00427

**Published:** 2019-03-18

**Authors:** Sara Schaefer, Kasper Arthur Hettinga, James Cullor, J. Bruce German, Bethany M. Henrick

**Affiliations:** ^1^Department of Food Science and Technology, Foods for Health Institute, University of California, Davis, Davis, CA, United States; ^2^Wageningen University and Research, Wageningen, Netherlands; ^3^School of Veterinary Medicine, University of California, Davis, Davis, CA, United States; ^4^Food Science and Technology Department, University of Nebraska Lincoln, Lincoln, NE, United States

**Keywords:** nutritional supplementation, dairy proteins, immune response, vaccines, UV-C treatment

In the original article, there was an error. The vaccine used in the trial is listed as Boostrix® (Merck Research Laboratory, Whitehouse Station, USA). The correct source of the DTaP vaccine used in this study is “Adacel® not “Boostrix®.”

A correction has been made to the **Materials and Methods**, **Human Samples**:

“Peripheral blood samples were collected at the time of enrollment (week 0) and serum was stored at −80°C until subsequent analysis. Participants were then randomized into two groups and provided with equal concentration and quantity of either dairy or soy supplement provided in powdered form in coded, single-serving bags. Both participants and researchers were blinded to the type of protein received. Participants were asked to consume two servings of protein powder (6 grams/packet) with 4 ounces of water or applesauce twice per day, with meals, for a total of 8 weeks. At week 4, participants were vaccinated with DTaP vaccine (Sanofi Pasteur Inc., Swiftwater PA). A 0.5-mL dose of Adacel® is formulated to contain 5 Lf of tetanus toxoid, 2.5 Lf of diphtheria toxoid, 8 μg of inactivated PT, 8 μg of FHA, and 2.5 μg of pertactin (69 kiloDalton outer membrane protein). Each 0.5-mL dose contains aluminum hydroxide as adjuvant (not more than 0.39 mg aluminum by assay), 4.5 mg of sodium chloride, ≤ 100 μg of residual formaldehyde, and ≤ 100 μg of polysorbate 80 (Tween 80). A second blood draw was obtained 4 weeks after vaccination (week 8) and serum collected for DTaP antibody analysis. The design and participant progression through the study is presented in Figure 1.”

The authors apologize for this error and state that this does not change the scientific conclusions of the article in any way. The original article has been updated.

